# Current Evidence on Nutrient Intakes and Infant Growth: A Narrative Review of Baby-Led Weaning vs. Conventional Weaning

**DOI:** 10.3390/nu16172828

**Published:** 2024-08-23

**Authors:** Kinzie L. Matzeller, Nancy F. Krebs, Minghua Tang

**Affiliations:** Department of Pediatrics, Section of Nutrition, University of Colorado Anschutz Medical Campus, Aurora, CO 80045, USA

**Keywords:** infants, complementary feeding, baby-led weaning, nutrition

## Abstract

Throughout infancy, changes in growth and development are observed, including changes in nutritional requirements; at approximately 6 months of age, when complementary feeding begins, breastmilk and/or formula are no longer the sole source of energy and nutrients. Little is known about the impact of complementary feeding (CF) approaches used during this time on infant nutrition and growth. Baby-led weaning (BLW) has continued to gain popularity over the last two decades, emphasizing the importance of examining the adequacy of different CF methods. This narrative review of 19 studies from January 2010 to April 2024 aims to discuss the differences between BLW and conventional weaning (CW). The definition of BLW varied across studies, and no standard definition has been established. Though no differences in energy were reported, macronutrient and micronutrient intakes were variable between approaches, including for micronutrients such as iron and zinc. Of the few studies with growth data, results comparing BLW and CW were conflicting. Differences were seen in the demographics of parents who chose to follow BLW and breastfeeding prevalence and duration prior to complementary feeding. Additional research is needed to understand the impacts of BLW and CW on nutrient intakes and growth to inform recommendations for infant complementary feeding approaches.

## 1. Introduction

Infancy is characterized by rapid growth and development changes, requiring dietary adaptation to support changing nutritional requirements. The World Health Organization (WHO) recently published an update to the ‘Guideline for complementary feeding of infants and young children 6–23 months of age’, which states that complementary feeding (CF) begins when breastmilk or milk formula alone no longer meets the nutritional requirements of infants, around 6 months of age [1,2]. The American Academy of Pediatrics (AAP) released guidance on CF, stating that CF refers to nutrient- and energy-containing solid and liquid foods, other than breastmilk or infant formula, provided to meet the increased needs of the infant [3,4,5]. The period of CF coincides with an important developmental period of feeding and eating behaviors and food preferences; thus, infants could benefit from consuming a variety of foods and textures during CF to potentially support this development [6]. Much of the literature has examined which types of foods to offer, including the current Dietary Guidelines for Americans (DGA), emphasizing the introduction of iron-rich, nutrient-dense foods while providing a variety of food groups to support healthy growth beyond infancy [7,8]. Despite the recognized importance of CF and the attention given to what infants should consume during this time, minimal research has evaluated the adequacy of popular methods of solid food introduction during infancy.

The approach of solid food introduction and the types of foods chosen by parents during this time play a critical role in the formation of long-term dietary patterns [9], and this decision is often influenced by conflicting information from healthcare providers, family members, and social media [10,11]. Other factors associated with CF practices include the maternal education level, mode of feeding (breastfeeding vs. formula feeding), and socioeconomic status [6,12,13]; while cultural beliefs and environments influence the CF approach, these factors will not be discussed in this work [14,15]. Since the commercialization of baby food production, parents and caregivers in the U.S. have followed a conventional weaning approach (CW), providing pureed foods in a parent-led approach; this has been the most common CF approach, with one study finding that almost 80% of infants consumed any sort of pureed or spoon-fed baby food [16]. During CF, caregivers are responsible for all feeding decisions for the infant [9]. As a potential outcome of the lack of dietary guidance targeting the period of CF, alternative approaches to CW have started to emerge and gain in popularity; one such approach is referred to as baby-led weaning (BLW) [6,9,17]. Emerging research on BLW has begun to examine if infant self-feeding models that emphasize responsive feeding practices are feasible and appropriate for infants during CF [6,18,19,20,21]. BLW focuses on offering table foods in finger-sized pieces rather than purees to promote infant self-feeding and limit purees and spoon-feeding [19,22]. Proponents of BLW state that this method encourages better self-regulation of intake, healthier long-term eating habits, and improved oral motor skills [6,17,22]; however, minimal research has been conducted on evaluating the nutritional adequacy of BLW in the growing infant.

The aim of this narrative review is to examine the evidence of BLW and CW as they relate to nutritional adequacy and growth. We will discuss current evidence for energy, macronutrient, and micronutrient intakes, observed growth patterns, and variations in the demographics who each follow a CF approach and suggest areas in which the research should be expanded to better support the development of guidelines and recommendations for complementary feeding.

## 2. Methods

### 2.1. Search Strategy and Data Source

The online databases of Medical Literature Analysis and Retrieval System Online (MEDLINE)/PubMed and Embase were searched to identify papers relevant to the research question. PubMed was identified as an appropriate database for a review as it allows full access to all datasets from a single search and can be used as a principal search system [23]. Search terms included “baby-led weaning” OR “baby led” OR “BLW”. Bibliographies of returned papers were manually searched for relevant articles. Articles were included if they were published between January 2010 and April 2024.

### 2.2. Inclusion and Exclusion Criteria

Original, full-length qualitative and quantitative studies and reviews of the BLW literature written in English were included. Studies that focused on nutrient intakes, growth, and demographics of parents and caregivers following BLW were included. As there is no standard definition of BLW, no definition of BLW was used as inclusion or exclusion criteria. Articles were excluded if the scope of the BLW work was outside the questions of this review. A total of 171 citations were returned from the MEDLINE/PubMed search and 154 citations from Embase; after screening for inclusion and exclusion criteria and manually searching bibliographies, 19 articles were included in the narrative review.

## 3. Defining Complementary Feeding Methods

The WHO, the AAP, and the DGA recommend introducing solid foods at approximately 6 months of age to complement a milk-based diet. The published guidelines provide insights into what foods to offer when beginning CF, focusing on the provision of animal-source foods, fruits and vegetables, pulses, nuts, and seeds [1,5,7]. The DGA provides additional recommendations, such as offering potentially allergenic foods, consuming a variety of food groups, foods high in iron and zinc, and avoiding added sugars and high sodium foods [7]. Despite recommendations for what to offer infants during CF, there is minimal research supporting how to offer these foods to infants; the choice often is made by caregivers based on the belief that the items chosen will improve their babies’ health [24]. In recent years, CW and BLW have emerged as two approaches to introducing solid foods, differing in how infants are offered solid foods during the period of CF.

### 3.1. Conventional Weaning

Traditionally, infant cereals have been amongst the first solid foods introduced to U.S. infants [25], though NHANES data show this is becoming less common over time [26]. Other first foods often include commercial baby food purees, with a reported 73–95% of U.S. infants [27] and 92% of German infants [28] consuming these products, though this number may be decreasing [26]; this indicates that spoon-feeding has been a popular method of CF amongst parents and caregivers. For the purpose of this review, CW will be used to refer to any method of CF not classified as BLW; for many studies in this review, infants were classified as CW if they were spoon-fed or consumed purees for more than 10% of their solid foods [12,29] or if they self-reported their infant as not following BLW [20,30,31,32].

### 3.2. Baby-Led Weaning

According to Google Trends, the search volume for baby-led weaning has been steadily increasing worldwide over the last two decades; in March 2024 alone, over 50,000 Google searches for “baby led weaning” were completed [33]. However, there is no standard definition of how to classify BLW in the literature. Brown and Lee (2011) were the first to apply a cutoff of ≤10% spoon-fed food and ≤10% pureed food as a classification for BLW [12], and this cutoff has been used by other researchers when defining BLW [29,34,35]. Dogan (2018) classified BLW similarly, with <10% of solid foods being purees or spoon-fed [18]. Many other studies have allowed parents and caregivers to self-report their feeding method as BLW [20,30,31,32,36]. There have been few randomized controlled trials; the Baby-Led Introduction to SolidS (BLISS) cohort instructed participants to follow a modified BLW method encouraging an adequate iron intake, which classified BLW as ≤10% of foods being parent-fed and at least 75% of foods being self-fed by the infant [20,21,34,37,38,39]. Though it has only recently gained popularity and has no formal definition, BLW is being used more frequently as an infant CF approach, as shown by an increase in the average consumption of finger foods and non-baby-food vegetables and meat by U.S. infants [26].

## 4. Results

A total of 18 publications were included in this review, which focused on the nutrient intakes and growth of BLW infants vs. CW infants. The characteristics and outcomes of the studies are detailed in Table 1.

### 4.1. Nutrient Intakes

#### 4.1.1. Energy Intake

One concern voiced by healthcare providers for infants following BLW is its nutritional adequacy compared to a more traditional approach using purees [46]. In multiple observational studies, there were no significant differences in reported absolute energy intake between BLW and CW infants at any time point between 6 and 12 months [20,21,29,34,38,40,41,43,45]; these findings were consistent between studies that used the BLISS definition [21,38,43], a ≤10% puree and spoon-feeding cutoff [20,29,34,40], and studies in which BLW was self-reported [17,41,45]. Only one study performed by Rowan, Lee, and Brown found that between 6 and 9 months, BLW infants consumed less energy than CW infants [40]; Cox found the opposite—at approximately 8 months of age, BLW infants were consuming more energy daily than CW [36]. While the majority of these studies weighed the infant’s dietary intake of solid foods and formula [20,21,38,39,40,41,45], there was no objective assessment of breastmilk intake, rather using estimations of breastmilk intake based on the infant’s age. Furthermore, energy intakes and subsequent nutrient intakes were reported as absolute values and were not controlled for infant weight (kcal/kg). Many of these studies had fewer than 100 participants, limiting the power to detect differences in nutrition and energy. Overall, current evidence on energy intakes between CW and BLW is inconsistent; additional research using measured breastmilk intake is required to assess the ability of BLW to provide adequate energy for infants relative to their weight.

#### 4.1.2. Macronutrient Intake

Carbohydrates are a major component of many complementary foods, regardless of preparation [47]. Despite the reported similar energy intakes between BLW and CW infants, differences are observed in macronutrient distribution between feeding approaches. In one study, BLW infants were reported to “like” carbohydrates more than their CW counterparts but were exposed to less carbohydrates than CW infants; in this study, BLW infants were significantly younger than CW infants, which may have contributed to differences seen in carbohydrate preference and exposure [30]. In the Bliss cohort, Erickson reports contradicting findings with higher consumption of grains and cereals in BLW infants than in CW [38]. Additionally, Rowan’s findings suggest that CW infants consumed more carbohydrates in the form of sugar than their BLW counterparts [40], while others have found there to be no difference in intake of carbohydrates [29] or from sugar [20], reflecting contradictions in the literature regarding typical carbohydrate consumption in both BLW and CW infants.

Dietary protein during complementary feeding plays an important role in establishing growth patterns and providing key micronutrients; lower consumption can impair normal growth, while high intakes have been asserted to promote excessive weight gain [48]. Provision of protein varies greatly between BLW and CW, with pureed meats and beans serving as the primary source for CW infants and bite-sized offerings in BLW. Protein intake varied in how it was reported across studies. Some measured protein intake in absolute quantities, while others reported the frequency of consumption of protein sources. Although some studies found that CW infants were offered and consumed more grams of protein daily than BLW infants [39,40], others showed that BLW infants had higher odds of consuming red meat [31] and were more likely to be offered protein than CW infants [41]; though not reported by all studies, Rowan included both animal and plant-based sources, but not dairy, toward daily protein intake [41]. Alpers measured protein intake using FFQs and 24 h diet recalls in a subset of the infants, finding no significant difference in protein intake between groups; however, CW infants were offered more dairy foods while BLW infants were offered more processed meats [29]. Protein intake was reported in total grams consumed daily in all studies and did not reference infant weight; future work should continue to examine protein in infancy, including comparisons between animal and plant-sourced protein.

During infancy, a sufficient amount of dietary fat is essential for increasing the energy density of the diet [49]; despite the risk of non-communicable diseases associated with saturated fat intake in adulthood, this is not observed in infants [50], further solidifying the importance of adequate fat intake in this population. Morison found that infants following BLW had higher total intakes of fat, saturated fat, and percent energy from fat compared to CW infants [20]; saturated fat accounted for 22% and 18% calories in the BLW and CW groups, respectively. Alpers reported no significant difference in saturated fat intake between methods [29]. The breakdown of dietary saturated fat sources was not reported in any study. The finding of higher total fat intake with BLW was consistent across other studies [20,29,34,38], with BLW infants reported to consume 6–8% more energy from fat than CW infants. One possible explanation for the observed increased saturated fat intake relates to breastmilk intake; BLW infants are more likely to be breastfed [12,18,20,21,38] and, therefore, may be receiving more saturated fat from human milk [51]. The long-term health effects of high saturated fat intake and sources of saturated fats in infancy are still widely unknown and warrant more examination.

Although energy intakes were similar between groups, current evidence suggests that macronutrient distribution differed between BLW and CW infants, with conflicting results regarding carbohydrates, protein, and fat. Breastmilk intake was estimated for all studies, and the classification of BLW varied between studies, which may contribute to conflicting findings across studies. Additional research is needed to determine the consequences, if any, of differences in macronutrient intake and to establish ideal intake ranges for infants following both CW and BLW as appropriate.

#### 4.1.3. Micronutrient Intake

Significant differences in micronutrient intakes have been identified between CW and BLW infants. The importance of providing iron-rich complementary foods during the first year of life, particularly in breastfed infants, cannot be overlooked. Because of the high risk of iron deficiency in breastfed infants starting between 4 and 6 months of age and the observed higher rates of breastfeeding with BLW, one concern raised regarding BLW is the ability to provide adequate iron to replenish stores [46], with an established EAR of 7 mg/day and RDA of 11 mg/day after 6 months of age [52]. In the BLISS cohort following modified BLW, both BLISS infants and control infants were offered similar amounts of iron daily (4.9 mg/day vs. 2.2 mg/day) [39]; a secondary analysis of iron intake in the BLISS cohort found similar results comparing BLW and CW at 7 months (3.0 mg/d vs. 2.7 mg/d) and 12 months (3.2 mg/d vs. 3.2 mg/d)43. Similarly, in a randomized trial by Dogan, BLW consumed similar iron to CW (7.97 mg/d vs. 7.8 mg/d) [18]. However, many other studies comparing BLW and CW found that infants following BLW consumed less iron, including Morison (1.6 mg/day vs. 3.6 mg/day) [20], Rowan (0.6 mg/day vs. 2.0 mg/day) [40], and Alpers (4.84 mg/day vs. 6.21 mg/day) [29]. Two studies reported higher intakes of iron in CW infants but did not provide values [19,20,29,34,40]. It is important to note that neither BLW nor CW infants met the recommended daily iron intake in any study.

Zinc-containing foods are another group highlighted in the DGA as important to include during complementary feeding [7] due to the decline in zinc content of breastmilk as the infant ages [53]. Zinc is implicated in immune function, physical growth, and cell division, emphasizing the importance of adequate zinc intake [54] of 2–3 mg/day [52]. Zinc intake was measured in a few studies, with Morison finding BLW infants consumed 0.7 mg less daily than CW infants (3.0 vs. 3.7 mg, respectively) [20], which is also reported by Pearce [34]. In the BLISS cohort, no difference in zinc intake was observed as both groups consumed 3.5 mg/day at 7 months and 4.4 mg/day at 12 months; however, the BLISS intervention was a modified form of BLW, limiting the generalizability of the result to all BLW infants [42]. As iron-rich foods often are also high in zinc, meat has been recommended as a first food during complementary feeding, regardless of CF method, to improve both iron and zinc intakes [55,56]

Nutrition guidelines across the world recommend limiting excessive salt during complementary feeding [57]; despite this recommendation, many commercially prepared foods and foods prepared by caregivers have high sodium content [58]. In infants, sodium is recommended to be limited, though there is no established upper limit. The Adequate Intake for infants older than 6 months is established to be 370 mg/d [59]. Some studies found that BLW infants consumed more sodium than CW infants; Alpers reported BLW infants to consume 529.1 mg/d compared to 375.5 mg/d in CW infants between 6 and 12 months [29]. Williams-Erickson reported higher intakes of sodium in BLW infants at 7 months (301 mg/day vs. 223 mg/day) [38], and Rowan reported slightly higher intakes at 6–9 months [40]. Only one study comparing sodium intake between groups reported no difference (235 mg/d in CW infants vs. 232 mg/d in BLW infants) [20]. One proposed explanation for this difference is the higher sodium levels of foods prepared for the family and given to BLW infants compared to conventionally prepared baby foods. For caregivers following BLW with their infants, foods should be salted at the table rather than during cooking to limit sodium intake for infants.

Vitamin B12 is important for nervous system function and red blood cell formation, with an RDA for infants of 0.4–0.5 mcg/day [60]. During CF, most of the dietary intake of vitamin B12 comes from the liquid diet. Vitamin B12 is present in breastmilk, though concentration decreases during the first months of lactation, and is a routine addition to formula [61,62]. Concentration in breastmilk varies [63], with one study reporting almost 20% of participant breastmilk samples were low in vitamin B12 [64]. Pearce found that CW infants had higher B12 intakes from complementary foods [34], with Morison finding similar results (0.2 mcg BLW vs. 0.5 mcg CW); Morison also reported lower intakes of zinc and iron with BLW, indicating that lower vitamin B12 intake may be attributed to different consumptions of animal-sourced foods [20]. Other studies found no significant difference in B12 intake [38,40], with all infants meeting the recommended daily intake. For infants following a plant-based complementary diet, as well as breastfed infants of vegan mothers, vitamin B12 intake should be prioritized independent of the CF approach.

Overall, results from the included studies reflect significant differences in micronutrient intake between BLW and CW, including iron, zinc, sodium, and vitamin B12. No significant differences were observed in demographic factors that may contribute to the observed differences, such as infant age, ethnicity, socioeconomic status, or maternal factors, except in two studies; these differences will be discussed separately [12,19]. Limitations of the current literature examining nutrient intake include estimation of breastmilk intake, self-reporting of food intake, variation in BLW classification, and small sample sizes.

### 4.2. Growth Patterns

A major concern raised by healthcare professionals regarding BLW is the possibility of growth faltering [65]. While BLW has been shown to provide similar energy to CW, it is important to examine growth pattern differences between CF approaches as dietary intake was self-reported. Some studies have found growth differences between BLW and CW infants; Dogan reported significantly higher weights in CW infants compared to BLW infants at 12 months of age (11.1 kg vs. 10.4 kg) and greater change in weight between 6 and 12 months. CW infants had higher rates of overweight using weight-for-length z-scores, though the statistical significance was not discussed. Between groups, BLW infants were exclusively breastfed for longer prior to introducing solid foods. As this difference was not controlled for during analysis, the mode of feeding may contribute to the differences observed in infant weight [18]. When looking at long-term differences in growth, Townsend found that BLW infants had a lower mean body mass index (BMI) rank in early childhood, which was closer to the median BMI, while CW infants had a mean percentile rank above the 50th percentile using both the NHS and CDC standards; weight-for-length z-scores were not reported [30]. Similarly, Brown reported that CW infants were significantly heavier than BLW infants as toddlers (12.86 kg vs. 11.79 kg) [44].

Other studies have found no significant differences between groups. In the BLISS cohort, no differences in BMI z-scores were observed at 12 and 24 months [21]. In agreement with the BLISS cohort, Alpers found no difference in weight-for-age or length-for-age percentiles at around 9 months of age [29]. While Jones did find differences in length between the groups, there were no differences in weight or weight gain velocity observed [32]. It should be noted that the collection of anthropometrics varied; weights were parent-reported [29,44], obtained from healthcare records [31], or measured by the researchers [18,21,32].

Despite conflicting evidence regarding growth differences associated with the CF approach, no growth faltering was reported in either group in any study; weight-for-length is the recommended method for monitoring growth of infants less than 24 months of age; however, most studies used BMI z-scores when reporting growth differences. Additional research should be added to the literature relating energy and nutrient intake with the growth of infants following CW or BLW to further clarify the adequacy of infant CF practices.

### 4.3. Other Differences between Baby-Led and Conventional Weaning

#### 4.3.1. Demographics

Demographic factors differ between groups. Following BLW was associated with higher socioeconomic status [12,19]. Brown found that mothers following a BLW approach with their infant were more likely to have a professional or managerial occupation or have a partner with this type of occupation [12], which aligns with the finding that mothers following a BLW approach had a higher education level than those following a CW approach [12,19]. Brown also found that mothers choosing BLW were less likely to return to work before their infant reached 12 months [12], with Komninou finding that more mothers of BLW infants were not in a paid occupation [66].

Information about infant CF practices was obtained from different sources dependent on the approach to CF followed. Caregivers and parents using BLW with their infant were more likely to obtain information about solid foods from social media and less likely to consult a healthcare provider for the information [31,66]. This suggests an opportunity for researchers to conduct further studies on BLW to ensure the promotion of evidence-based recommendations from both media and healthcare providers.

#### 4.3.2. Breastfeeding

When comparing modes of feeding, multiple studies found differences between CW and BLW infants. Infants following BLW were more likely to be breastfed at the time of starting complementary feeding [34,40]; additionally, BLW was associated with longer breastfeeding duration [12,18,20,21,38], which ranged from 1 week [18,38] to 8 weeks longer in BLW infants [20]. Furthermore, infants were more likely to be exclusively breastfed (EBF) for at least 6 months [29,31,45] when compared to infants following CW. Alpers found that 64% of infants following BLW were EBF for 6 months compared to 32% of CW infants [29], which was similar to the findings of Cameron (53% in BLW vs. 28% in CW) [45].

#### 4.3.3. Timing and Type of Complementary Food Introduction

Consequently, longer exclusive breastfeeding duration correlates to the age of complementary food introduction; BLW infants were introduced to solid foods later than their CW counterparts [12,21,38]. Age of introduction ranged from 1.1 [40] to 4.4 weeks [21] earlier in CW infants than BLW infants. Only one study found no significant difference in the age of complementary food introduction [31]. The delayed introduction of complementary foods corresponds to the findings that BLW infants are more likely to be EBF for 6 months, reflecting that mothers of BLW infants are delaying the introduction of solid food intake longer than mothers of CW infants.

Many studies found that CW infants were more likely to have received commercially prepared baby foods than BLW infants [12,40,45]; these findings are unsurprising, as commercially prepared baby foods are mostly found as purees and require spoon-feeding. Many studies also found that BLW infants were more likely to regularly consume foods also being consumed by the family rather than separate meals [12,20,45].

## 5. Discussion

Despite the growing popularity of BLW as a feeding approach, this review reflects a lack of conclusive evidence for nutrient intakes and growth outcomes between the two approaches, confirming what has been discussed in other reviews of CF approaches [6,19]. The evidence suggests that BLW provides similar energy compared to CW, while the macronutrient composition of the diet may vary between groups. In addition, micronutrient intake differences may exist between BLW and CW, though the evidence is inconclusive in many nutrients such as iron, zinc, and vitamin B12. Dietary intake data, including measurement of breastmilk intake, should be prioritized to provide a clearer picture of the intake of these nutrients associated with different CF approaches. Evidence supporting growth differences between BLW and CW infants is conflicting, though it is important to note that anthropometrics were not always obtained by researchers and may be inaccurate; future work should examine growth and dietary intake concurrently to evaluate the outcomes of these approaches. Differences exist in who follows BLW and CW; BLW infants were more likely to be EBF for longer, leading to a later introduction of solid foods. Demographic factors may influence the choice of CF approach, including higher maternal education and higher socioeconomic status in parents following BLW. Parents and caregivers choosing to follow BLW are more likely to obtain their knowledge from social media rather than healthcare providers; with the evolving digital-age environment, healthcare providers should stay attentive to non-traditional feeding approaches gaining in popularity to provide evidence-based guidance in response to information found on social media.

The current evidence discussed in this review has several limitations. Most studies assessing the adequacy and outcomes of BLW as an approach to CF are observational, cross-sectional, or short-term. As there is no standard definition of BLW in the literature, the classification of BLW varies greatly between studies and limits the ability to compare outcomes between studies. None of the included studies used an objective measure of energy intake controlling for infant weight, and very few studies included anthropometric data, emphasizing the need for research evaluating growth outcomes between CF approaches. Additionally, breastmilk intake was not measured in any study; this may have influenced the reported macronutrient, micronutrient, and energy intakes between groups, as BLW infants were more likely to be breastfed and had a longer duration of breastfeeding. Test weighing of nursed breastmilk sessions, in addition to the weighing of bottles, would provide this information in future work.

## 6. Conclusions

The period of CF is an important time to establish long-term feeding behaviors, food preferences, and growth trajectories. While there are potential benefits of BLW that have been discussed previously, much remains unclear regarding the adequacy of BLW to support nutrient intakes and growth during CF. Despite the findings of similar energy intakes between feeding approaches, the impact of BLW on macronutrient and micronutrient intakes remains inconclusive, with conflicting growth outcomes across studies. Assessing the adequacy of BLW is difficult, particularly due to the lack of a standard definition of BLW in research and in practice; the establishment of one such definition would add consistency and reliability to future research examining BLW and other CF methods. The literature is generally in agreement that BLW and CW provide similar energy and that BLW may have potential benefits for long-term outcomes; however, without conclusive evidence regarding macronutrient and micronutrient intakes and growth outcomes, the ability to comprehensively recommend BLW as a CF approach is limited at this time. Additional research with large sample sizes, comprehensive dietary analysis, and researcher-obtained anthropometric measurements should be emphasized to improve the quality of the evidence base. Future research should examine the differences in growth and macronutrient and micronutrient intake while finding strategies to mitigate dietary gaps that may be associated with the CF method to assist in the development of evidence-based recommendations.

## Figures and Tables

**Table 1:** BLW and CW Study Characteristics.

Reference	N	Study Type	Infant Age Range (Months)	Classification of BLW	Study Focus	Outcomes
Cox [36] (2024)	625	Observational	6.5–9.5	Self-report	Energy intake and growth	- BLW infants consumed more energy than CW infants at 8 months (150 kJ/day)
Bocquet [19] (2022)	N/A	Narrative Review	N/A	N/A	Describe and assess BLW in the literature	- BLW infants consumed less iron than CW infants - BLW was associated with a higher socioeconomic status and higher maternal education
Rowan [40] (2022)	71	Observational	6–12	Self-report (<10% purees/spoon-feeding)	Energy and nutrient intake	- Between 6 and 9 months, BLW infants consumed less energy (623 kcal/d) than CW infants (742 kcal/d) - Between 6 and 9 months, BLW infants consumed more sodium and less protein (13.9 g/d vs. 18.9 g/d g) and iron (1.1 mg/d vs. 3.0 mg/d) than CW infants - BLW infants were more likely to be breastfed when CF started (88.4% vs. 57.8%)
Pearce [34] (2021)	96	Cross-sectional	6–12	Self-report (<10% purees/spoon-feeding)	Adherence, food group exposure, nutrient intake	- BLW infants more likely to be breastfed (77.8% vs. 41.7%) - CW infants had higher intakes of iron, zinc, vitamin D, vitamin B12 between 6 and 8 months - BLW infants had higher percent energy intake from fat and saturated fat between 6 and 8 months
Jones [32] (2020)	269	Secondary analysis (SHIFT)	3–12	Self-report	Growth	- No differences in weight or BMI between BLW and CW - CW infants were significantly longer than BLW infants (z-score 0.11 vs. −0.42)
Alpers [29] (2019)	134	Cross-sectional	6–12	Self-report (<10% purees/spoon-feeding)	Energy and nutrient intake	- BLW was associated with exclusive breastfeeding (64% vs. 32%) and later CF introduction (5.8 vs. 5.5 months) - No difference in energy intake - BLW infants consumed less iron (4.84 mg/d vs. 6.21 mg/d) but more fat from food (15.9 g/d vs. 10.2 g/d) and sodium than CW infants (529.1 mg/d vs. 375.5 mg/d)
Rowan [41] (2019)	180	Cross-Sectional	6–12	Self-report (<10% purees/spoon-feeding)	Dietary intake	- BLW infants were more likely to be exposed to protein at 6–8 months - No differences in exposure to iron-rich foods
Daniels [42] (2018)	206	Secondary analysis (BLISS RCT)	7–12	BLISS Modified BLW ^a^	Zinc intake	- No differences in zinc intake at 7 months (3.5 mg/d vs. 3.5 mg/d) and 12 months (4.4 mg/d vs. 4.4 mg/d)
Daniels [43] (2018)	206	Secondary analysis (BLISS RCT)	7–12	BLISS Modified BLW ^a^	Iron intake	- No differences in iron intake between BLW and CW infants at 7 months (3.0 mg/d vs. 2.7 mg/d) or 12 months (3.2 mg/d vs. 3.2 mg/d) - No differences in energy intake between BLW and CW infants at 7 months (2862 kJ/d vs. 2996 kJ/d) or 12 months (3573 kJ/d vs. 3623 kJ/d)
Dogan [18] (2018)	280	RCT	5–12	BLISS Modified BLW ^a^	Food preferences, intake, and growth	- CW infants had heigher weights at 12 months (10.4 kg vs. 11.1 kg) - No significant differences in iron intake (7.97 mg/d vs. 7.9 mg/d) - BLW infants were breastfed for longer than CW infants
Fu [31] (2018)	876	Cross-sectional	6–36	Self-report	Food fussiness and weight	- BLW infants had higher odds of consuming red meat at 6–7 months (68% vs. 52%) - Caregivers of BLW infants were more likely to obtain information about CF from social media (58% vs. 19%) - BLW infants had a longer duration of exclusive breastfeeding (4.6 months vs. 4 months)
Morison [20] (2016)	206	Cross-sectional	6–8	Self-report	Energy and nutrient intake	- No differences in absolute energy intake in BLW and CW infants (2800 kJ/d vs. 2897 kJ/d) - BLW infants consumed more fat (36 g/d vs. 33 g/d), saturated fat (17 g/d vs. 14 g/d), and percent energy from fat (48% vs. 42%) - BLW infants consumed less iron (1.6 mg/d vs. 3.6 mg/d), zinc (3 mg/d vs. 3.7 g/d), and vitamin B12 (0.2 mcg/d vs. 0.5 mcg/d) - BLW was associated with longer breastfeeding duration (22.2 weeks vs. 14.4 weeks) while more CW infants were introduced to solid foods before 6 months (96% vs. 50%)
Williams Erickson [38] (2018)	206	Secondary analysis (BLISS RCT)	7–12	BLISS Modified BLW ^a^	Energy and nutrient intake	- No significant differences in absolute energy intake between BLW and CW infants at 7 months (2951 kJ/d vs. 2831 kJ/d) or 12 months (3484 kJ/d vs. 3373 kJ/d) - BLISS infants consumed more total fat (35 g/d vs. 33.2 g/d), and sodium (301 mg/d vs. 223 mg/d) at 7 months - No significant difference in vitamin B12 intake in BLW (0.6 mcg/d) and CW infants (0.5 mcg/d)
Taylor [21] (2017)	206	Secondary analysis (BLISS RCT)	6–24	BLISS Modified BLW ^a^	Growth and risk of overweight	- No differences between BLW and CW in BMI z-scores (0.44 vs. 0.2) or prevalence of overweight (15.1% vs. 6%) at 12 months
Cameron [39] (2015)	23	Pilot intervention	5.5–9	BLISS Modified BLW ^a^	BLISS pilot study	- BLISS infants were offered more grams of protein (14.7% of energy) than BLW infants (10.8% of energy) - No significant differences in iron intake between BLISS infants (4.9 mg/d) and BLW infants (2.2 mg/d)
Brown [44] (2013)	298	Longitudinal	6–24	Self-report (<0% purees/spoon-feeding)	Satiety responsiveness and growth	- CW infants (12.86 kg) were significantly heavier than BLW infants (11.79 kg) at 18–24 months - CW infants had higher rates of overweight (19.2%) at 18–24 months than BLW infants (8.1%)
Cameron [45] (2013)	199	Cross-sectional	6–12	Self-report	Feeding practices	- BLW infants were more likely to be exclusively breastfed for 6 months than CW infants (53% vs. 21%)
Townsend [30] (2012)	155	Case-controlled sample	20–78	Self-report	Food preferences and growth	- BLW infants preferred carbohydrates, whereas CW infants preferred sweet foods - Increased incidence of underweight in BLW infants (9.5%) than CW infants (1.6%) using CDC percentiles - Increased incidence of obesity in CW infants (11.1%) than BLW (1.6%) using CDC percentiles
Brown [12] (2011)	655	Qualitative	6–12	Self-report (<10% purees/spoon-feeding)	Demographics of BLW	- BLW was associated with higher socioeconomic status, higher maternal education, and professional/managerial operation - BLW was associated with longer breastfeeding duration (18.2 weeks vs. 11.7 weeks) and later CF introduction

^a^ BLISS Modified BLW weaning method provided education to parents. All foods were offered in a form following BLW while caregivers received education to address concerns for iron intake, choking, and growth faltering. N/A refers to review articles in which sample size was not discussed.

## References

[1] World Health Organization Guideline for Complementary Feeding of Infants and Young Children 6–23 Months of Age. https://iris.who.int/bitstream/handle/10665/373358/9789240081864-eng.pdf?sequence=1.

[2] Jonsdottir O., Thorsdottir I., Hibberd P., Fewtrell M., Wells J., Palsson G., Lucas A., Gunnlaugsson G., Kleinman R. (2012). Timing of the Introduction of Complementary Foods in Infancy: A Randomized Controlled Trial. Pediatrics.

[3] Scientific Advisory Council on Nutrition (2018). Feeding in the First Year of Life. https://assets.publishing.service.gov.uk/media/5b48c28aed915d481c04f1e2/SACN_report_on_Feeding_in_the_First_Year_of_Life.pdf.

[4] Fewtrell M., Bronsky J., Campoy C., Domellöf M., Embleton N., Fidler Mis N., Hojsak I., Hulst J.M., Indrio F., Lapillonne A. (2017). Complementary Feeding: A Position Paper by the European Society for Paediatric Gastroenterology, Hepatology, and Nutrition (ESPGHAN) Committee on Nutrition. J. Pediatr. Gastroenterol. Nutr..

[5] Kleinman R., Greer F., Krebs N. (2020). Complementary Feeding. Pediatric Nutrition Handbook.

[6] Boswell N. (2021). Complementary Feeding Methods–A Review of the Benefits and Risks. Int. J. Environ. Res. Public Health.

[7] USDA Dietary Guidelines for Americans, 2020–2025. https://www.dietaryguidelines.gov.

[8] DiMaggio D.M., Cox A., Porto A.F. (2017). Updates in Infant Nutrition. Pediatr. Rev..

[9] Birch L.L., Doub A.E. (2014). Learning to eat: Birth to age 2 y. Am. J. Clin. Nutr..

[10] Spurlock K., Deave T., Lucas P.J., Dowling S. (2023). Parental engagement with complementary feeding information in the United Kingdom: A qualitative evidence synthesis. Matern. Child Nutr..

[11] Garcia A.L., Looby S., McLean-Guthrie K., Parrett A. (2019). An Exploration of Complementary Feeding Practices, Information Needs and Sources. Int. J. Environ. Res. Public Health.

[12] Brown A., Lee M. (2011). A descriptive study investigating the use and nature of baby-led weaning in a UK sample of mothers. Matern. Child Nutr..

[13] Mekonen E.G., Zegeye A.F., Workneh B.S. (2024). Complementary feeding practices and associated factors among mothers of children aged 6 to 23 months in Sub-saharan African countries: A multilevel analysis of the recent demographic and health survey. BMC Public Health.

[14] Nandagire W.H., Atuhaire C., Egeineh A.T., Nkfusai C.N., Tsoka-Gwegweni J.M., Cumber S.N. (2019). Exploring cultural beliefs and practices associated with weaning of children aged 0–12 months by mothers attending services at Maternal Child Health Clinic Kalisizo Hospital, Uganda. Pan Afr. Med. J..

[15] Vázquez-Frias R., Ladino L., Bagés-Mesa M.C., Hernández-Rosiles V., Ochoa-Ortiz E., Alomía M., Bejarano R., Boggio-Marzet C., Bojórquez-Ramos M.C., Colindres-Campos E. (2023). Consensus on complementary feeding from the Latin American Society for Pediatric Gastroenterology, Hepatology and Nutrition: COCO 2023. Rev. Gastroenterol. Mex..

[16] Haszard J.J., Heath A.M., Katiforis I., Fleming E.A., Taylor R.W. (2024). Contribution of Infant Food Pouches and Other Commercial Infant Foods to the Diets of Infants: A Cross-sectional Study. Am. J. Clin. Nutr..

[17] Brown A., Jones S.W., Rowan H. (2017). Baby-Led Weaning: The Evidence to Date. Curr. Nutr. Rep..

[18] Dogan E., Yilmaz G., Caylan N., Turgut M., Gokcay G., Oguz M.M. (2018). Baby-led complementary feeding: Randomized controlled study. Pediatr. Int..

[19] Bocquet A., Brancato S., Turck D., Chalumeau M., Darmaun D., De Luca A., Feillet F., Frelut M.L., Guimber D., Lapillonne A. (2022). “Baby-led weaning”–Progress in infant feeding or risky trend?. Arch. Pediatr..

[20] Morison B.J., Taylor R.W., Haszard J.J., Schramm C.J., Williams Erickson L., Fangupo L.J., Fleming E.A., Luciano A., Heath A.L. (2016). How different are baby-led weaning and conventional complementary feeding? A cross-sectional study of infants aged 6–8 months. BMJ Open.

[21] Taylor R.W., Williams S.M., Fangupo L.J., Wheeler B.J., Taylor B.J., Daniels L., Fleming E.A., McArthur J., Morison B., Erickson L.W. (2017). Effect of a Baby-Led Approach to Complementary Feeding on Infant Growth and Overweight: A Randomized Clinical Trial. JAMA Pediatr..

[22] Cameron S.L., Heath A.L., Taylor R.W. (2012). How feasible is Baby-led Weaning as an approach to infant feeding? A review of the evidence. Nutrients.

[23] Gusenbauer M., Haddaway N.R. (2020). Which academic search systems are suitable for systematic reviews or meta-analyses? Evaluating retrieval qualities of Google Scholar, PubMed, and 26 other resources. Res. Synth. Methods.

[24] Afflerback S., Carter S.K., Anthony A.K., Grauerholz L. (2013). Infant-feeding consumerism in the age of intensive mothering and risk society. J. Consum. Cult..

[25] Klerks M., Bernal M.J., Roman S., Bodenstab S., Gil A., Sanchez-Siles L.M. (2019). Infant Cereals: Current Status, Challenges, and Future Opportunities for Whole Grains. Nutrients.

[26] Duffy E.W., Kay M.C., Jacquier E., Catellier D., Hampton J., Anater A.S., Story M. (2019). Trends in Food Consumption Patterns of US Infants and Toddlers from Feeding Infants and Toddlers Studies (FITS) in 2002, 2008, 2016. Nutrients.

[27] Briefel R.R., Reidy K., Karwe V., Devaney B. (2004). Feeding infants and toddlers study: Improvements needed in meeting infant feeding recommendations. J. Am. Diet. Assoc..

[28] Kersting M., Alexy U., Sichert-Hellert W., Manz F., Schöch G. (1998). Measured consumption of commercial infant food products in German infants: Results from the DONALD study. Dortmund Nutritional and Anthropometrical Longitudinally Designed. J. Pediatr. Gastroenterol. Nutr..

[29] Alpers B., Blackwell V., Clegg M.E. (2019). Standard v. baby-led complementary feeding: A comparison of food and nutrient intakes in 6–12-month-old infants in the UK. Public Health Nutr..

[30] Townsend E., Pitchford N.J. (2012). Baby knows best? The impact of weaning style on food preferences and body mass index in early childhood in a case-controlled sample. BMJ Open.

[31] Fu X., Conlon C.A., Haszard J.J., Beck K.L., von Hurst P.R., Taylor R.W., Heath A.M. (2018). Food fussiness and early feeding characteristics of infants following Baby-Led Weaning and traditional spoon-feeding in New Zealand: An internet survey. Appetite.

[32] Jones S.W., Lee M., Brown A. (2020). Spoonfeeding is associated with increased infant weight but only amongst formula-fed infants. Matern. Child Nutr..

[33] Trends G. “Baby Led Weaning”. https://trends.google.com/trends/explore?date=all&q=baby%20led%20weaning.

[34] Pearce J., Langley-Evans S.C. (2022). Comparison of food and nutrient intake in infants aged 6–12 months, following baby-led or traditional weaning: A cross-sectional study. J. Hum. Nutr. Diet..

[35] Watson S., Costantini C., Clegg M.E. (2020). The Role of Complementary Feeding Methods on Early Eating Behaviors and Food Neophobia in Toddlers. Child Care Pract..

[36] Cox A.M., Taylor R.W., Haszard J.J., Beck K.L., von Hurst P.R., Conlon C.A., Te Morenga L.A., Daniels L., McArthur J., Paul R. (2024). Baby food pouches and Baby-Led Weaning: Associations with energy intake, eating behaviour and infant weight status. Appetite.

[37] Daniels L., Heath A.L., Williams S.M., Cameron S.L., Fleming E.A., Taylor B.J., Wheeler B.J., Gibson R.S., Taylor R.W. (2015). Baby-Led Introduction to SolidS (BLISS) study: A randomised controlled trial of a baby-led approach to complementary feeding. BMC Pediatr..

[38] Williams Erickson L., Taylor R.W., Haszard J.J., Fleming E.A., Daniels L., Morison B.J., Leong C., Fangupo L.J., Wheeler B.J., Taylor B.J. (2018). Impact of a Modified Version of Baby-Led Weaning on Infant Food and Nutrient Intakes: The BLISS Randomized Controlled Trial. Nutrients.

[39] Cameron S.L., Taylor R.W., Heath A.L. (2015). Development and pilot testing of Baby-Led Introduction to SolidS--A version of Baby-Led Weaning modified to address concerns about iron deficiency, growth faltering and choking. BMC Pediatr..

[40] Rowan H., Lee M., Brown A. (2022). Estimated energy and nutrient intake for infants following baby-led and traditional weaning approaches. J. Hum. Nutr. Diet..

[41] Rowan H., Lee M., Brown A. (2019). Differences in dietary composition between infants introduced to complementary foods using Baby-led weaning and traditional spoon feeding. J. Hum. Nutr. Diet..

[42] Daniels L., Taylor R.W., Williams S.M., Gibson R.S., Samman S., Wheeler B.J., Taylor B.J., Fleming E.A., Hartley N.K., Heath A.M. (2018). Modified Version of Baby-Led Weaning Does Not Result in Lower Zinc Intake or Status in Infants: A Randomized Controlled Trial. J. Acad. Nutr. Diet..

[43] Daniels L., Taylor R.W., Williams S.M., Gibson R.S., Fleming E.A., Wheeler B.J., Taylor B.J., Haszard J.J., Heath A.-L.M. (2018). Impact of a modified version of baby-led weaning on iron intake and status: A randomised controlled trial. BMJ Open.

[44] Brown A., Lee M.D. (2015). Early influences on child satiety-responsiveness: The role of weaning style. Pediatr. Obes..

[45] Cameron S.L., Taylor R.W., Heath A.L. (2013). Parent-led or baby-led? Associations between complementary feeding practices and health-related behaviours in a survey of New Zealand families. BMJ Open.

[46] Cameron S.L., Heath A.L., Taylor R.W. (2012). Healthcare professionals’ and mothers’ knowledge of, attitudes to and experiences with, Baby-Led Weaning: A content analysis study. BMJ Open.

[47] Abeshu M.A., Lelisa A., Geleta B. (2016). Complementary Feeding: Review of Recommendations, Feeding Practices, and Adequacy of Homemade Complementary Food Preparations in Developing Countries–Lessons from Ethiopia. Front. Nutr..

[48] Kittisakmontri K., Lanigan J., Wells J.C.K., Fewtrell M. (2020). The Impact of Dietary Protein in Complementary Foods on Infant Growth and Body Composition in a Population Facing the Double Burden of Malnutrition: Protocol for a Multicenter, Prospective Cohort Study. JMIR Res. Protoc..

[49] Michaelsen K.F., Grummer-Strawn L., Bégin F. (2017). Emerging issues in complementary feeding: Global aspects. Matern. Child Nutr..

[50] Agostoni C., Caroli M. (2012). Role of fats in the first two years of life as related to later development of NCDs. Nutr. Metab. Cardiovasc. Dis..

[51] Delplanque B., Gibson R., Koletzko B., Lapillonne A., Strandvik B. (2015). Lipid Quality in Infant Nutrition: Current Knowledge and Future Opportunities. J. Pediatr. Gastroenterol. Nutr..

[52] Institute of Medicine (US) Panel on Micronutrients (2001). Dietary Reference Intakes for Vitamin A, Vitamin K, Arsenic, Boron, Chromium, Copper, Iodine, Iron, Manganese, Molybdenum, Nickel, Silicon, Vanadium, and Zinc.

[53] Krebs N.F., Westcott J.E., Culbertson D.L., Sian L., Miller L.V., Hambidge K.M. (2012). Comparison of complementary feeding strategies to meet zinc requirements of older breastfed infants. Am. J. Clin. Nutr..

[54] Azevedo-Silva T.R., Vivi A.C.P., Fonseca F.L.A., Lebrão C.W., Strufaldi M.W.L., Sarni R.O.S., Suano-Souza F.I. (2023). Association of serum and erythrocyte zinc levels with breastfeeding and complementary feeding in preterm and term infants. J. Dev. Orig. Health Dis..

[55] Krebs N.F., Westcott J.E., Butler N., Robinson C., Bell M., Hambidge K.M. (2006). Meat as a first complementary food for breastfed infants: Feasibility and impact on zinc intake and status. J. Pediatr. Gastroenterol. Nutr..

[56] Hawthorne K.M., Castle J., Donovan S.M. (2022). Meat Helps Make Every Bite Count: An Ideal First Food for Infants. Nutr. Today.

[57] Cribb V.L., Warren J.M., Emmett P.M. (2012). Contribution of inappropriate complementary foods to the salt intake of 8-month-old infants. Eur. J. Clin. Nutr..

[58] Maalouf J., Cogswell M.E., Bates M., Yuan K., Scanlon K.S., Pehrsson P., Gunn J.P., Merritt R.K. (2017). Sodium, sugar, and fat content of complementary infant and toddler foods sold in the United States, 20151, 2, 3. Am. J. Clin. Nutr..

[59] Stallings V.A., Quirk M., Oria M., National Academies of Sciences Engineering and Medicine (U.S.), Committee to Review the Dietary Reference Intakes for Sodium and Potassium, National Academies of Sciences Engineering and Medicine (U.S.) Food and Nutrition Board (2019). Dietary Reference Intakes for Sodium and Potassium.

[60] Institute of Medicine (US) Standing Committee on the Scientific Evaluation of Dietary Reference Intakes and its Panel on Folate OhBV, and Choline (1998). Dietary Reference Intakes for Thiamin, Riboflavin, Niacin, Vitamin B.

[61] Greibe E., Nexo E. (2016). Forms and Amounts of Vitamin B12 in Infant Formula: A Pilot Study. PLoS ONE.

[62] Dror D.K., Allen L.H. (2018). Vitamin B-12 in Human Milk: A Systematic Review. Adv. Nutr..

[63] Henjum S., Manger M., Hampel D., Brantsæter A.L., Shahab-Ferdows S., Bastani N.E., Strand T.A., Refsum H., Allen L.H. (2020). Vitamin B12 concentrations in milk from Norwegian women during the six first months of lactation. Eur. J. Clin. Nutr..

[64] Pawlak R., Vos P., Shahab-Ferdows S., Hampel D., Allen L.H., Perrin M.T. (2018). Vitamin B-12 content in breast milk of vegan, vegetarian, and nonvegetarian lactating women in the United States. Am. J. Clin. Nutr..

[65] Pesch M.H., Levitt K.J., Danziger P., Orringer K. (2021). Pediatrician’s Beliefs and Practices Around Rapid Infant Weight Gain: A Qualitative Study. Glob. Pediatr. Health.

[66] Komninou S., Halford J.C.G., Harrold J.A. (2019). Differences in parental feeding styles and practices and toddler eating behaviour across complementary feeding methods: Managing expectations through consideration of effect size. Appetite.

